# Presence and Health Risks of Obsolete and Emerging Pesticides in Paddy Rice and Soil from Thailand and China

**DOI:** 10.3390/ijerph17113786

**Published:** 2020-05-27

**Authors:** Naranun Khammanee, Yanling Qiu, Nipapun Kungskulniti, Anders Bignert, Yuan Meng, Zhiliang Zhu, Zebene Lekew Teffera

**Affiliations:** 1 Key Laboratory of Yangtze River Water Environment (Ministry of Education), College of Environmental Science and Engineering, Tongji University, Shanghai 200092, China; nuau_naranun@hotmail.com (N.K.); 1810054@tongji.edu.cn (Y.M.); zzl@tongji.edu.cn (Z.Z.); 2 College of Environmental Science and Engineering, UNEP-Tongji Institute of Environment for Sustainable Development, Tongji University, Shanghai 200092, China; tzebenel@yahoo.com; 3 Faculty of Public Health, Mahidol University, Bangkok 10400, Thailand; nipapun123@yahoo.com; 4 Center of Excellence on Environmental Health and Toxicology (EHT), Bangkok 10400, Thailand; 5 Swedish Museum of Natural History, 10691 Stockholm, Sweden; anders.bignert@nrm.se

**Keywords:** organochlorine pesticides, organophosphorus pesticides, paddy field, rice, soil, health risks

## Abstract

Organochlorine (OCPs) and organophosphorus pesticides (OPPs) have been intensively applied in rice paddy field farming to control pest infestation and increase the yield. In this study, we investigated the presence of organochlorine and organophosphorus pesticides in paddy rice and soil from rice plantations in Thailand and China. According to concentration and distribution of OCPs, the most abundant OCPs residues in rice and soil from Thailand and China were dichlorodiphenyltrichloroethane and hexachlorocyclohexanes. The OPPs of methidathion, carbophenothion, chlorpyrifos, and diazinon were common to Thailand and China in both types of samples. The detection frequency of multiple types of these pesticides was greater than 50% of total samples. The relative concentration of some OPPs residues in rice and soil from Thailand and China were significantly different from each other (*p* < 0.0083), whereas, no significant difference was observed for the relative concentration of OCPs residues in rice and soil from both countries, except for HCHs (*p* < 0.05). Bioaccumulation factors of OCPs between rice and soil samples indicated that OCPs and OPPs in soil could accumulate in rice. The carcinogenic and non- carcinogenic risks of OCPs and OPPs seem to be in the safe range as recommended by the European Union.

## 1. Introduction

The rapid economic expansion and population growth in developing countries has led to an increased demand of rice to ensure sufficient food for consumption and storage for safety. To increase rice productivity, the usage of pesticides has increased. Total global pesticide production increased from 1 to 3 million tons between the 1960s and 2000s [1]. In southern Asia, Thailand is one of the top importers of pesticides from China, accounting for 25% of exported Chinese pesticides from 2016 to 2018 [2]. China is the world’s largest exporter of pesticides, at a value of 4.8 billion US dollars, representing 14% of exported pesticides worldwide [3]. The total pesticide consumption of China dramatically rose from 1.28 million tons in 2001 to 1.81 million tons in 2014, approximately 16 to 86-fold greater than those of Thailand [4]. Pesticides have played a crucial role in the growth and stability of developing countries for several decades. Their main purpose is to control pest infestation in large areas of agricultural land and malaria vector control in households. The dominant targets of pesticides exhibited found in several countries including Thailand and China were *Nilaparvat lugens* (Delphacidae), *Sogatella furcifera* (Cicadellidae), *Nephotettix nigropictus* (Cicadellidae), *Stenchaetothrip biformis* (Thripidae), *Parnara guttata* (Hesperiidae), *Nymphula depunctalis* (Pyralidae) [5]. Farmers have indiscriminately applied a variety of insecticides in paddy fields to significantly increase yield per unit.

Organochlorine pesticide (OCPs) is a major group of pesticides used by local farmers in developing countries due to cheap cost and inadequate credit facilities during the past several decades [6]. The application of dichlorodiphenyltrichloroethane (DDT) in agriculture and malaria repellents has been extensive in Thailand since the early 1950s and was phased out in 1994. The cessation of hexachlorocyclohexanes (HCHs), drins (aldrin, dieldrin, endrin, isodrin), and chlordanes (oxychlordane, α-, β- chlordane) application in Thailand was mandated in 2000 [7]. Prior to Thailand, the use of DDTs and HCHs in China was formally prohibited in 1983 [8]. The higher residue levels of these pesticides in rice and soil samples from Thailand and China were inconsistent with the pesticide control policy in the two countries [5]. The great impact of illegal OCPs is still affecting their residues in environments for a long time, and hence threatening ecological and human health.

Nowadays, many kinds of organophosphorus pesticides (OPPs) have been substituted for OCPs due to their broad spectrum of activity, lower persistence, and lower toxicity. However, these pesticides are persistent, lipophilic, hydrophobic, and bioaccumulative. Soil is a major absorber of these pesticides that inadvertently, eventually enter higher trophic levels organisms by desorption, dissolution, diffusion, transpiration, and accumulation. These pesticides can act on the acetylcholinesterase enzyme that is reported to cause nerve malfunction and disruption of the reproductive system [9,10]. As biomarkers of OPPs in the human body, acetylcholinesterase (AChE) and butyrylcholinesterase (BChE) levels in blood samples from agricultural farmers at San Pa Tong and Mae Taeng district, Northern Thailand were lower than those in nonfarm workers [9,11] Besides, its mutagenicity, teratogenicity, and carcinogenicity have an adverse effect on humans. The intensive use of OPPs has been proved to pose a threat to children in rural area of Zhejiang, China [12]. Environmental state and behavior of pesticides in rice and soil have become challenging issues in developing countries. The main objectives of the present study were (1) to identify the occurrence and behavior of OCP and OPP residues in rice and soil samples from Thailand and China, (2) to elucidate the correlation between the concentration and patterns of target pesticides in rice and soil from these two countries, (3) to assess the correlation between the concentration of target pesticides and total organic carbon (TOC) in soil from these two countries, and (4) to evaluate the health risks of consuming rice from these two countries.

## 2. Materials and Methods

### 2.1. Study Area and Sample Collection

**Figure 1:**
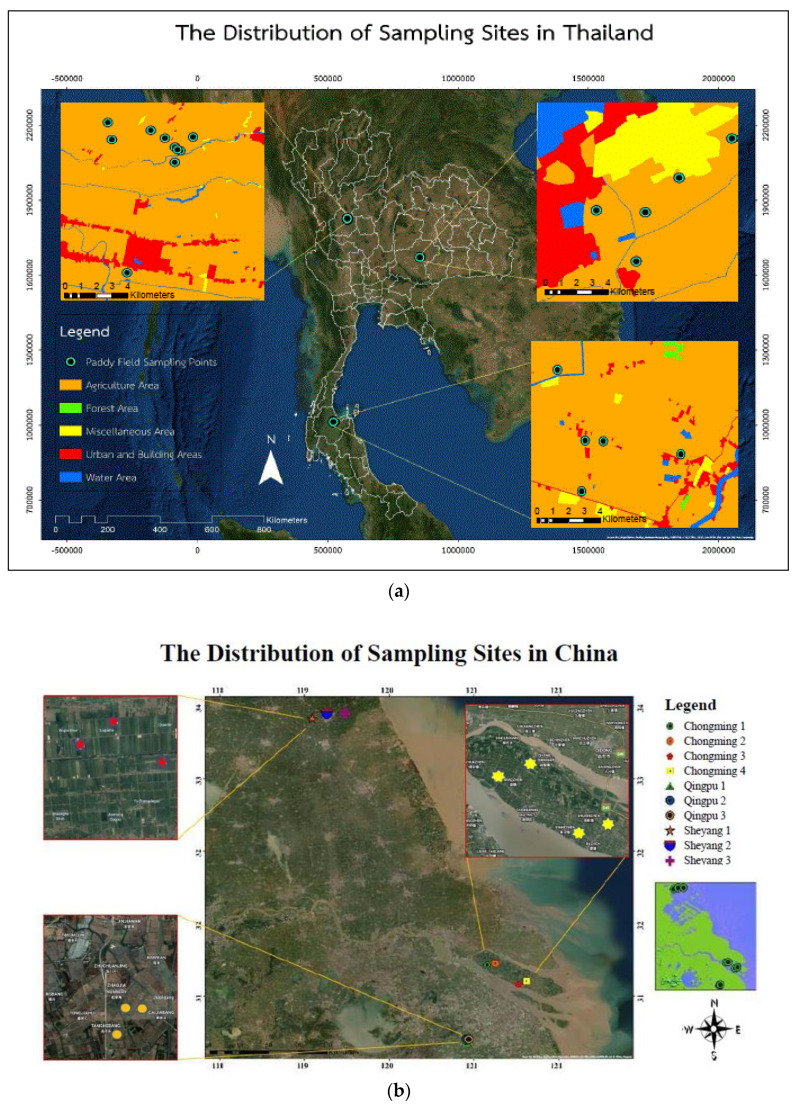
The distribution of sampling sites: (**a**) rice and soil sampling sites in Thailand, (**b**) rice and soil sampling sites in China.

A total number of 20 rice (*Oryza sativa*) and 20 soil samples were taken from paddy fields located in different parts of Thailand. The locations were as follows: northern Thailand—Kamphaengphet province (*n* = 20) (16°48′ N, 99°70′ E), northeastern Thailand—Nakorn Ratchasima province (*n* = 10) (15°08′ N, 102°28′ E), and southern Thailand—Surat Thani province (*n* = 10) (9°15′ N, 99°19′ E) (Figure 1a). A total number of 10 rice (*Oryza sativa*) and 10 soil samples were taken from the Yangtze River Delta (YRD), China. Samples were collected from Qingpu, Shanghai (*n* = 6) (31°09′ N, 120°89′ E), Chongming Island, Shanghai (*n* = 8) (31°36′ N, 121°29′ E), and Sheyang, Jiangsu province (*n* = 6) (33°44′ N, 120°13′ E) (Figure 1b).

Rice and surface soil samples from both Thailand and China were collected simultaneously after harvesting during 2015–2016 (Table 1). For each sampling site, five subsamples of soil or rice consisting of one center and four corners were pooled and homogenized to form a composite sample. Each subsample site was taken randomly and covered approximately 20 × 30 m^2^. A 20 cm deep soil was taken for each subsample site by using steel spades. All samples were packed into plastic bags and stored in a refrigerator at −20 °C in the laboratory until analysis. Prior to residue extraction, rice and soil samples were dried at room temperature, grinded, and sieved through a sieve with 100 mesh number (0.149 mm of diameter). 

### 2.2. Chemicals and Reagents

The analytical grade of chemicals and materials were obtained from ANPEL Laboratory Technologies Co., Ltd., Shanghai, China. The pesticide standards of OCPs and OPPs were purchased from AccuStandard, New Haven, USA. The mixture of 22 OCPs standards contains hexachlorocyclohexanes (HCHs: α-, β-, γ-,δ-HCH), hexachlorobenzene (HCB), drins (aldrin, dieldrin, endrin, isodrin), chlrodanes (CHLs: oxychlordane, α-, β- chlordane), endosulfans (α-, β-endosulfan), dichlorodiphenyltrichloroethane (DDTs: *o,p’*-DDE, *p,p’*-DDE, *o,p’*-DDD, *p,p’*-DDD, *o,p’*-DDT, *p,p’*-DDT), methoxychlor, and mirex. The mixture of 15 OPPs standards contains azinphos methyl, carbophenothion, chlorpyrifos, chlorpyrifos methyl, diazinon, dichlorvos, ethion, fenitrothion, fonofos, malathion, methidathion, methyl parathion, parathion, phosalone, and pirimiphos-methyl. CB-53 and CB-200 as surrogate standard, and CB-189 and pentachloronitrobenzene (PCNB) as internal standard for OCPs were also supplied by AccuStandard. Tri-n-butylphosphate (TnBP-d27) as surrogate standard, and triphenyl phosphate (TPP-d15) as internal standards for OPPs were purchased from Dr. Ehrenstorfer, Augsburg, Germany. 

### 2.3. Analytical Methods of OCPs

The extractions of target OCPs, in homogenized rice and soil samples (5.00 ± 0.05 g) were transferred into a 50 mL centrifuge tube containing 30 ng CB-53 and 40 ng CB-200 as a surrogate standard prior to extraction. The rice and soil samples were extracted with 30 mL of normal hexane: acetone (8:2, v/v) by shaking vigorously for 1 min and sonicating by using ultrasonication for 10 min thrice. The supernatants were pooled and concentrated by a rotary vacuum evaporator at 40 °C, and then purged under a gentle nitrogen gas. The rice extracts were purified by gel permeation chromatography (GPC) with Bio-Beads SX-3 columns, which is supplied by ANPEL Laboratory Technologies (Shanghai), Shanghai, China. The soil extracts were loaded into 2 g of alumina column packed with 1 g of Na_2_SO_4_, and then interacted with copper overnight into the tube to remove sulfur. The further purified rice and soil extracts were cleaned up using a florisil column (500 mg). Finally, purified eluant was filtered through a 0.22 μm membrane filter and finally added 20 ng of internal standard (CB-189 and PCNB) prior to GC-ECD analysis.

### 2.4. Analytical Methods of OPPs

The extraction and cleanup of target OPPs was slightly modified from the QuEChERS (quick, easy, cheap, effective, rugged, and safe) method [13]. Rice samples (5.00 ± 0.05 g) were extracted with 30 mL acetonitrile (ACN) thrice. The rice extracts were vigorously shaken by vortex at a speed of 2500 rpm for 1 min and centrifuged at 2000 rpm for 10 min. The supernatant was dehydrated by passing through 5 g of NaCl and then concentrated to be 1 mL using a rotary vacuum evaporator. For dispersive solid phase extraction (d-SPE), 500 mg of MgSO_4_, 100 mg of primary secondary amine (PSA), and 50 mg of graphite carbon black (GCB) were added to the centrifuged supernatant in a 10 mL tube. After that, the supernatant (1 mL) was further cleaned up with NH_2_ and GCB cartridges. 

Soil samples (5.00 ± 0.05 g) were extracted thrice with 30 mL ethyl acetate (EtOAc). The extract was vigorously shaken by vortex at 2500 rpm for 1 min and centrifuged at 2000 rpm for 10 min. For dispersive solid phase extraction (d-SPE), 4 g of MgSO_4_ and 1 g of NaCl were added to the supernatant in a 10 mL tube. The supernatant was further cleaned up with florisil and GCB cartridges. Both rice and soil aliquot were evaporated up to 0.5 mL by a gentle nitrogen gas. Prior to analysis, the purified eluant was filtered through a 0.45 μm PTFE filter.

### 2.5. Instrumental Analysis

The quantitation and qualification of the target OCPs were conducted by using Agilent 7890A gas chromatograph equipped with a ^63^Ni electron capture detector has been obtained from Agilent Technologies Co.LTD., Santa Clara, CA, USA. The conditions of the mentioned instrument were well established [14]. Oven temperature program was initially set at 80 °C and held for 2 min, then increased to 195 °C at the rate of 10 °C min^−1^. The increased temperature ramped to 230 °C at the rate of 3 °C min^−1^, finally ramped to 310 °C at the rate of 10 °C min^−1^, and held for 5 min. 

The target OPPs were analyzed using a gas chromatography-tandem mass spectrometry (GC-MS/MS) equipped with a triple quadrupole mass spectrometer quantum that has been sourced from Thermo Fisher Scientific S.p.A., Strada Rivoltana, Milan, Italy. Separation of the compounds was carried out on a thermo scientific TG-5ms GC column (30 m × 0.25 mm i.d., 0.25 um film thickness), that has been sourced from Thermo Fisher Scientific Co.Ltd., Waltham, Massachusetts, USA, and mass spectrometer in the electron impact ionization mode (EI) at 70 eV. The oven temperature was programmed from the initial temperature of 40 °C, increased to 200 °C at a rate of 20 °C min^−1^ and held for 2 min, finally ramped to 280 °C at 8 °C min^−1^ and held for 3 min. The injector temperature of 250 °C in splitless mode and 2 μL of injection volume were set. Helium was used as a carrier gas at a constant flow rate of 1.0 mL min^−1^. 

### 2.6. Quality Assurance and Quality Control (QA/QC)

The method blank and duplicate samples were checked in every batch of 20 samples. Quantitation was performed using the internal calibration method based on an eight-point calibration curve for individual OPPs and OCPs. For each pesticide, the standard curve had a wide linear range from 1 to 500 ngg^−1^ with the correlation coefficient (r^2^) greater than 0.99. The QC samples were spiked with 100 ng of tri-n-butyl phosphate-d27 for OPPs, 30 ng of CB-53 and 40 ng of CB-200 for OCPs as a surrogate standard, which were in the acceptable recovery range from 76% to 110%. The limit of detection (LOD) and the limit of quantification (LOQ) of target OCPs were calculated by a signal-to-noise ratio of 3 and 10, ranging from 0.02 to 0.76 and 0.06 to 2.6 ngg^−1^, respectively. The method detection limits (MDLs) and method quantification limits (MQL) of OPPs ranged from 0.02 to 0.76 and 0.06 to 2.6 ng g^−1^, respectively. As a further quality assurance to validate the method, the certified reference materials (CRMs) of soil for OPPs (SQCO-021) and OCPs (SQCO-003) were purchased from NSI Lab Solution Inc., North Carolina, USA (*n* = 3). The measurement results of the target ∑_10_OCPs and ∑_6_OPPs in CRMs were all in the acceptance limits with exception of diazinon, aldrin, cis-endosulfan, and cis-chlordane. All results were expressed on a dry weight basis.

### 2.7. Statistical Analysis

The distribution of OCPs and OPPs in soil from Thailand and China has been visualized by using ArcGIS (Ver. 10.0; Esri (Thailand) Co.,Ltd., Bangkok, Thailand). All Statistical analysis and drawing were performed by using SPSS software (SPSS Inc, Chicago, IL, USA). Principal component analysis (PCA) was carried out in order to study potential differences in patterns of OCP and OPP concentrations relative to the sum of concentrations between Thailand and China and between rice and soil. Hotelling’s T^2^-test was conducted to check if differences in patterns were significant. The correlation coefficient (r^2^) of OCPs and OPPs in rice and soil were established based on the Pearson correlation. In order to check for disproportionate influence of extreme values, the Mann–Kendall coefficient (τ) was compared with the Pearson correlation coefficients. The relationship between the residue levels of OCPs and OPPs and physicochemical property (TOC) were analyzed by using the Mann–Kendall trend test. The level of significance was set to 5% and Bonferroni adjusted for repeated tests when necessary. The chronic hazard indices (HI) measures the potential risk of adverse health effects from a mixture of pollutants derived from the sum of target hazard quotient. Target hazard quotient was calculated by ratio of the estimate daily intake rate (EDI) divided by the acceptable daily intake (ADI). Acute health risks (aHI) was calculated by ratio of the estimate of short-term intake (ESTI) divided by the acute reference dose (ARfD) [15]. The estimate of pesticide intake in the diet was compared to maximum residue limit (MRL) for these pesticides, which are rectified by China [16], Thailand [17], and EU standard [18]. The formulation mentioned above, if the value is greater than one, there is a chance of non-carcinogenic effects. If the value is less than one, it is assumed to be safe for risk of non-carcinogenic effects. The incremental lifetime cancer risks (ILCRs) [19].

## 3. Results

### 3.1. OCP and OPP Residues in Paddy Rice

The total concentration of 22 OCP residues (∑_22_OCPs) in rice samples from Thailand ranged from 34.04 to 2061 ng g^−1^ (108 ng g^−1^ of median). The decreasing order of median concentration (ng g^−1^) of OCPs residues in rice samples from Thailand was DDTs (32.3) > HCHs (30.2) > drins (21.4) (Table S1). Except for cis-endosulfan, the detection frequencies of all 21 OCPs in rice samples from Thailand were over 58%. The total concentration of 15 OPP residues (∑_15_OPPs) in rice samples from 3 regions of Thailand ranged from 5.14 to 171 ng g^−1^ (17.0 ng g^−1^ of median). The concentration (ng g^−1^) of OPP residues in rice samples from Thailand was in a decreasing order of methidathion (7.26) > carbophenothion (5.43) > diazinon (1.22) (Table S2). The detection frequencies of 9 out of 15 types of OPP residues in rice samples from Thailand were over 58%. 

The ∑_22_OCPs of rice samples from China ranged from 37.4 to 393. ng g^−1^ with a median of 44.0 ng g^−1^. The residue level (ng g^−1^) of OCPs in rice samples from China was in a decreasing order of DDTs (21.5) > HCHs (8.06) > CHLs (4.55), as shown in Table S1. The detection frequencies of 20 OCPs in all rice samples from China were over 60%, with exception of trans-endosulfan and HCB. The median ∑_22_OCPs in rice samples from Thailand was approximately 2.5 times higher than that in China. The residue levels of DDTs and HCHs in rice samples from Thailand and China were dramatically higher than other OCPs, due to their intensive application and strong persistence. Among all samples, the highest residue level of DDTs (257 ng g^−1^) was found in rice from southern Thailand. For China, the highest HCHs concentration (251 ng g^−1^) was found in rice from Sheyang.

The ∑_15_OPPs in rice samples from China ranged from 20 to 118 ng g^−1^ with a median of 27.6 ng g^−1^. The detected concentration (ng g^−1^) of individual OPPs was in a decreasing order of methidathion (19.8) > carbophenothion (2.48) > chlorpyrifos (1.79), as shown in Table S2. The detection frequencies of 9 out of 15 types of OPP residues in rice samples from China were over 50%. The median residue level of ∑_15_OPPs in rice from Thailand and China were comparable, with a level of 29.2 and 29.1 ngg^−1^, respectively. The highest ∑_15_OPPs among all rice samples was detected from northern Thailand, with a median of 171 ng g^−1^. Among 15 OPPs, methidathion, chlorpyrifos, carbophenothion, and diazinon were abundant in rice from both of Thailand and China. Methidathion had the highest residue levels in rice samples from southern Thailand and Sheyang, China, which were 7.26 and 19.8 ng g^−1^, respectively. 

### 3.2. OCP and OPP Residues in Paddy Soil

The ∑_22_OCPs in soil samples from Thailand ranged from 3.37 to 135 ng g^−1^ with a median of 52.1 ng g^−1^. The residue level (ng g^−1^) of OCPs in soil samples from Thailand followed the order of DDTs (16.8) > drins (11.5) > HCHs (8.1) (Table S3). The detection frequencies of 21 OCPs in soil samples from Thailand were over 65% with the exception of endosulfans. The median ∑_22_OCPs residue in soil samples from China was roughly 2 times higher than that in Thailand. The residue levels of HCHs, DDTs, and drins in soil samples from both Thailand and China were much higher than other OCPs. The ∑_15_OPPs in soil samples from 3 regions of Thailand ranged from 2.05 to 58.7 ng g^−1^ with a median of 3.87 ng g^−1^. The concentration (ng g^−1^) of OPP residues in soil samples from Thailand was in a decreasing order of methidathion (1.30) > pirimiphos-methyl (0.91) > azinphos methyl (0.70), as shown in Table S4. The detection frequencies of 10 out of 15 types of OPPs in soil samples from Thailand were over 55%.

The ∑_22_OCPs in soil samples from China ranged from 55.2 to 407 ng g^−1^ with a median of 122 ng g^−1^. The order of OCP concentration (ng g^−1^) in soil samples from China was DDTs (40.6) > HCHs (37.2) > drins (21.2) (Table S3). The detection frequencies of OCPs in all soil samples from China were all over 70%. The ∑_15_OPPs in soil samples from China ranged from 2.91 to 22.2 ng g^−1^ with a median of 3.09 ng g^−1^. The OPP residues concentration (ng g^−1^) in soil from China was in a decreasing order of methidathion (1.10) > diazinon (1.09) > chlorpyrifos (0.24), as shown in Table S4. The detection frequencies of most of the 15 OPPs in soil samples from China were over 50%, except for dichlorvos, methyl-parathion, ethion, carbophenothion, and phosalone. The median residue level of ∑_15_OPPs in soil from Thailand was slightly higher than those of China.

Total organic carbon (TOC) plays an important role in determination of the distribution and behaviors of OCPs and OPPs. In the present study, the TOC content in soil samples from Thailand and China ranged from 4.83% to 8.9%, and 5.79% to 7.01%, respectively.

### 3.3. Potential Health Risks of OCPs and OPPs in Paddy Rice

On the basis of the non-carcinogenic risk of OCP exposures, the highest chronic hazard indices (HIs) for Thai male and female children were 14.9 and 15.3 at southern, and 14.5 and 15.0 at northeastern regions, respectively. The highest HIs of OCPs exposures for Chinese male and female adults were 3.08 and 2.70 in Sheyang, and 1.76 and 2.00 in Chongming, respectively. For all three pathways (ingestion, dermal, and inhalation), the highest values of incremental lifetime cancer risks (ILCRs) of the target OCPs for Thai and Chinese children were negligible (less than 1). Among pathways of ingestion, dermal absorption, and inhalation, the greatest ILCRs of exposure to OCPs in rice through dermal absorption were sensitive to Thai male children, with a value of 1.988 × 10^−6^, it is assumed to be safe for risk of non-carcinogenic effects. No cancer slope factor was available for the calculation of ILCRs of OPPs through the ingestion and dermal pathways.

## 4. Discussion

### 4.1. Presence of OCPs in Paddy Rice and Soil

β-HCH was the first or second dominant HCH composition in rice and soil from both Thailand and China. In general, γ-HCH and α-HCH can be transformed into β-HCH in soil, which is the most resistant to biodegradation among the four HCH isomers. For the six target DDTs, *p,p’*-DDE and *o,p’*-DDE were found the major congeners in rice and soil from both Thailand and China. DDT parent compound can be transformed into DDE through biodegradation under aerobic conditions, which could be accumulated by crops from contaminated soil through the uptake of root exudate [20]. The concentration of HCHs in the present study was relatively higher than rice products reported in other studies, e.g., Jiangsu province, China (29 ng g^−1^) [21], Shanghai and Yixing, China (lower than 0.05 ngg^−1^) [22], and India (10 ngg^−1^) [23]. Whereas, the present study was apparently lower than the level of HCHs in rice from the Bueng Boraphet wetland, Thailand [24]. The concentrations of DDTs (29 ngg^−1^) in rice and its bran products from the Jiangsu province, China were higher than those in the present study [21]. The average of DDT residue contained 2 to 40 ng g^−1^ in rice grain from India was similar of those in the present study [25]. The sum of the concentrations of OCPs in rice samples in the present study were comparable to rice grains from Dehradun, Punjab Province, Pakistan [26] and Chenab, Pakistan [27]. 

The HCH and DDT concentrations in soil of this study were comparable to the agricultural soil from Minh Dai and Hoang Liet of northern Vietnam, with average levels of HCHs (47 and 58 ng g^−1^) and DDTs (28 and 17 ng g^−1^), respectively [28]. In the present study, the HCB level in soil samples from Thailand was similar to the agricultural soil from Kathmandu, Nepal (1.56 ng g^−1^) [29], but relatively lower than soils from Wuhan, China (up to 17.8 ng g^−1^) [30]. 

In general, an isomeric ratio of *p,p’*-DDT/(*p,p’*-DDE + *p,p’*-DDD) greater than 1 suggests fresh input of technical DDT, while a ratio less than 1 suggests historical use of DDT. Results in this study showed that all soil samples from Thailand and 90% of total soil samples from China originated from historical use of DDTs. For technical grade HCHs, the α-/γ-HCH ratio varies from 4 to 7, indicating that the transformation of the parent compounds over time occurred. If this ratio is near zero, it usually suggests the use of lindane, which contains almost pure γ-HCH [31,32,33]. In this study, the ratio of α-/γ-HCH in all soil samples from Thailand and China ranged from 1 to 2.54, suggesting no obvious new input of technical HCHs and lindane. A ratio of cis-chlordane/trans-chlordane greater than 1 indicates aged chlordane, whereas a ratio of lower than 1 indicates fresh chlordane. In the present study, 95% of soil samples from Thailand and 90% of soil samples from China indicated aged chlordane. The technical endosulfan product has the α-/β-endosulfan ratio of approximately 2.33. If the ratio is less than 2.33, it normally suggests a historical use of endosulfans. In the present study, 80% of the soil samples from Thailand and 100% of the soil from China suggested the historical usage of endosulfans. 

According to the Chinese National Soil Quality Standard [34], the concentration of DDTs in 15% of total soil from Thailand and 40% of total soil from China exceeded the target value of 50 ngg^−1^, this could be considered as low and medium contamination, respectively. The concentrations of HCHs in 30% of total soil from Thailand and 30% of total soil from China exceeded the target value of 50 ngg^−1^, this could be considered as low contamination. 

### 4.2. Presence of OPPs in Paddy Rice and Soil

Nowadays, OPP pesticides are widely applied in paddy rice, as substitutes for OCPs. The highest ∑_15_OPPs found in soil samples from northern Thailand was 58.6 ng g^−1^, which coincides with the maximum residues of ∑_15_OPPs in paddy rice from same region. Methidathion, chlorpyrifos, and diazinon were the predominant OPPs detected in soils from Thailand and China. The above results corroborate the findings of Snyder and Ni [35], the largest number of supplies in China consist of chlorpyrifos (409), followed by diazinon (92) and methidathion (12). The structures of these OPPs (chlorpyrifos, methidathion, pirimiphos-methyl, and diazinon) are more complex than other types of OPP residues such as aliphatic or phenol organophosphorus groups. Further, these compounds are strongly accumulated in paddy rice and soil due to high Kow and Koc, low vapor pressure, low Henry’s law constant, and low water solubility [36]. Pesticide residues in soil compared to plants are lower, or in some cases, non-existent, which might be due to volatilization, leaching, surface run-off, soil properties, and migration of pesticide residue into invertebrates’ bodies and plants [37]. Generally, the compatibility of pesticides and compartment of paddy rice and soil are mainly dependent on sophistication processes such as absorption, translocation, and metabolism to alter pesticide structure [38]. 

The concentration of diazinon in rice in the present study was dramatically lower than diazinon in rice farming (58.4 ng g^−1^) and rice-fish farming paddies (117 ng g^−1^) from Can Tho, Vietnam [39]. The average of diazinon in rice samples (31.9 mg/kg) from Anzali International Wetland basin, Iran [40] was far higher than the present study. Both in dehusked and rough rice grain at 30 to 40 days pre-harvest intervals from Philippines [41], no residues of chlorpyrifos, methyl parathion, and diazinon were detected, which was considerably lower than the present study. 

The OPPs residues in soil from previous studies, the lower mean residue level of malathion (below detection limit) and higher mean residue level of chlorpyrifos (1.49 ng g^−1^) from the topsoil of Yangtze river basin, China [42] were relatively higher than in the present study. The results from this study were dramatically lower than the residue of chlorpyrifos in paddy fields of soil samples from Nanning and Guangxi province, China, which were 510 and 630 ng g^−1^, respectively [43], and from Indonesia with a range of 137 to 363 ng g^−1^ for malathion and 11 to 63 ng g^−1^ for chlorpyrifos, which was due to a large amount and high frequency of application of the relevant pesticides by the local farmers [44]. 

### 4.3. Bio-Concentration Factor of OCPs and OPPs between Paddy Rice and Soil

The highest bioaccumulation factor (BCF) of OCPs between rice and soil samples from Thailand was drins (5.90), followed by DDTs (3.58) and HCHs (3.17). The BCF of mirex and methoxychlor was 1.46 and 1.08, respectively, the highest BCF in rice from China. The largest BCF of OPPs from China and Thailand were methidathion and phosalone, with a value of 29.9 and 22.5, respectively. It is known that plants can uptake contaminants from soil through root exudates and finally translocate them to the seeds at the mature stage. In addition, spray drift of pesticide application to foliar directly can cause direct absorption by the plants [45]. The difference of the BCF for a total of 22 OCPs and 15 OPPs mainly depends on the concentration of these pesticides in soil, the interaction of water-soluble pesticides and the root exudates, and the amount of fatty content which retains pesticides in rice grain [38]. The results from the present study were in accordance with the BCF of drins in rice from Beung Boraphet wetland, Thailand were higher than 0.5 [24]. The BCFs of OCPs in nut and soil from Heilongjiang, Jilin, Hebei, Hubei, Sichuan, Jiangxi, and Yunnan, China were similar to the present study, which ranged from 1.10 to 16.5 [46]. Endosulfans in rice samples from southeastern China showed the BCF varying from 1.19 to 5.88, that was relatively higher than the present study [47]. Carrot-accumulated lindane from soil was shown by the BCF in peel (884), body (450), tap root (976), and shoot (53), supporting that lindane from soil could dramatically translocate into other parts of the plant [38]. 

### 4.4. Patterns and Relative Concentrations of OCP and OPP between Paddy Rice and Soil

**Figure 2:**
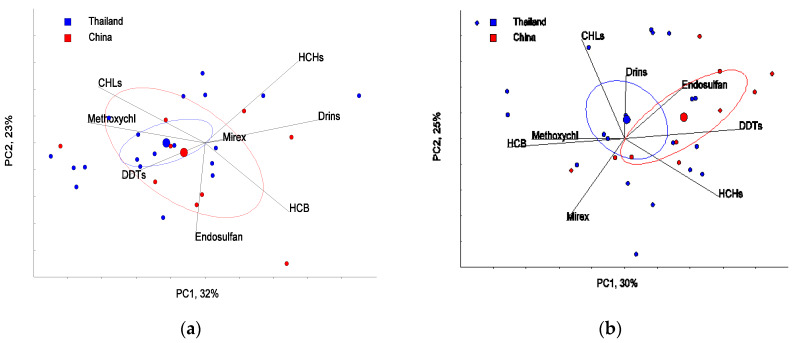
Principal component analysis (PCA): (**a**) PCA of rice, (**b**) PCA of soil from Thailand and China exposure to organochlorine pesticides (OCPs); chlordane (CHLs), methoxychlor (methoxychl), hexachlorobenzene (HCB), mirex, drins, hexachlorocyclohexanes (HCHs), dichlorodiphenyltrichloroethane (DDTs), endosulfans.

Principal component analysis (PCA) was conducted to determine the underlying structure of the variable and degradation behavior of target OCPs and OPPs in paddy fields. The classifications of 4 major groups among rice and soil from both Thailand and China were explained using the first two principal components (PC1, PC2). PC1 versus PC2 showed loading for individual OCPs in rice and soil both from Thailand and China which explained 55% of the total variation (Figure 2a,b). However, the patterns of OCP residues in rice and soil between Thailand and China showed no significant differences in the OCP patterns (Hotelling’s T^2^-test). This result can be explained with reference to all OCP residues having similar physicochemical properties such as high hydrophobicity, low water solubility and vapor pressure, bioaccumulation, and persistence in the environment. The underlying structure of the variable and degradation behavior of target OCPs between rice and soil in paddy fields were similar. This result agreed with OCPs in agricultural soil of Shanghai, China [48]. 

**Figure 3:**
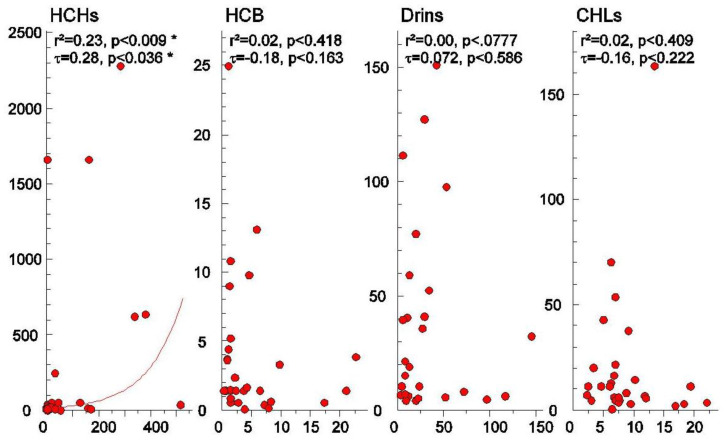
Concentration of individual OCPs (HCHs, HCB, drins, CHLs) between rice (*Y*-axis) and soil (*X*-axis) in Thailand and China. * represented by significant at the 95% confidence interval (*p* < 0.05).

**Figure 4:**
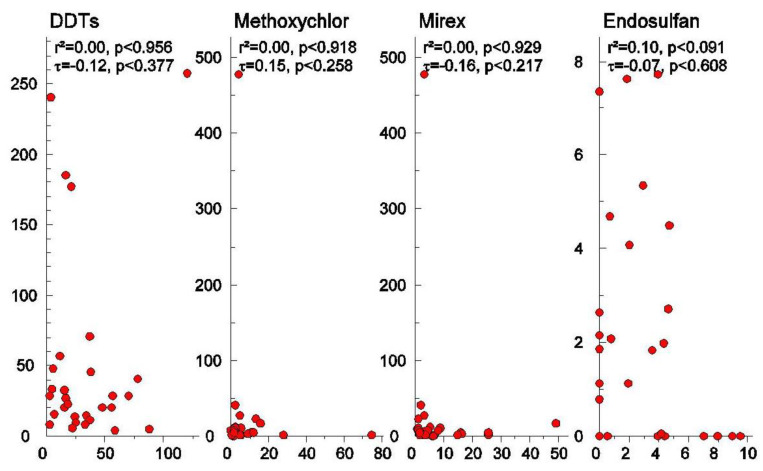
Concentration of individual OCPs (DDTs, methoxychlor, mirex, endosulfan) between rice (*Y*-axis) and soil (*X*-axis) in Thailand and China.

The relative concentration of rice (*Y*-axis) and soil (*X*-axis) in Thailand and China with a coefficient of determination (r^2^) based on the Pearson correlation and the non-parametric of the Mann–Kendall coefficient (τ) are illustrated, as presented in Figure 3 and Figure 4. None of the 22 OCPs residues showed a significant correlation between rice and soil neither from Thailand nor from China, except for HCHs (*p* < 0.05), which showed a positive significant correlation both with Pearson correlation and the Mann–Kendall association test (one extreme value in samples from the northeast and south of Thailand makes the Pearson correlation inappropriate). This is consistent with the results of the PCA analyses. 

**Figure 5:**
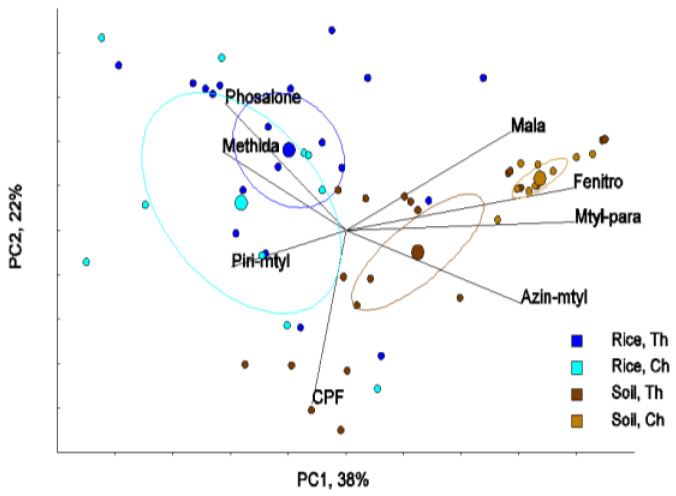
Principal component analysis (PC1, PC2) of organophosphorus (OPPs) in rice and soil from Thailand and China: malathion (mala), fenitrothion (Fenitro), methyl parathion (Mtyl-para), azinphos methyl (Azin-mtyl), chlorpyrifos (CPF), phosalone (phosalone), methidathion (methida), pirimiphos methyl (Piri-mtyl), rice of Thailand (Rice, Th), rice of China (Rice, Ch), soil of Thailand (Soil, Th), soil of China (Soil, Ch).

The first two, PC1 and PC2, showed loading for individual OPPs in rice and soil, both from Thailand and China which explained 55% of the total variation. The patterns of OPP residues in both rice and soil between Thailand and China showed a significant different pattern, whereas Hotelling’s 95% confidence ellipses for methidathion in rice was overlapped between China and Thailand (Figure 5). In the PCA, the relative concentrations are projected down to the PC-axis. Potential differences of the relative concentration among rice and soil from Thailand and China (4 groups) were tested with Hotelling’s T^2^-test, and the achieved *p*-values were compared with the Bonferroni adjusted alpha (*p* < 0.0083). The results showed that the relative concentrations of the OPPs-pattern in rice from China was significantly different from those from Thailand (*p* < 0.0011). In addition, the relative concentration in soil from China was significantly different from soil in Thailand (*p* < 0.0035), as illustrated in Table 2. 

### 4.5. Correlation among TOC and Individual OCPs and OPPs

The relationship between total organic carbon (TOC) and individual OCPs and OPPs in soil from Thailand and China were investigated by Kendall’s tau correlation coefficient (τ) and the 95% confident interval. The results showed no significant correlation between TOC and individual OCPs. Poor correlation between individual OCPs and TOC may be due to land use, particle size of soil, composition of organic carbon, and physicochemical characteristics of OCPs [43]. Furthermore, some other factors from the ambient environment could have an influence. For soil from Thailand, a significant positive correlation between TOC content and diazinon (*p* = 0.019, τ = 0.389), azinphos-methyl (*p* = 0.021, τ = 0.385), phosalone (*p* = 0.033, τ = 0.384) were exhibited. A significant positive correlation between TOC content and pirimiphos-methyl (*p* = 0.047, τ = 0.528) in soil from China was shown. This suggests that these OPPs probably originated from similar sources. 

### 4.6. Potential Health Risks of OCPs and OPPs in Paddy Rice

In spite of the common presence of OPP residues in rice, the non-carcinogenic risk of OPPs exposure to Thai and Chinese farmers and consumers was negligible. However, OCP exposures may pose potential health problems to these groups. The OCPs non-carcinogenic risk by hazard index (HIs) exceeded the acceptable level for all groups of consumers from the studied areas, where the group of drins was the main contributor for health risk. Children are the most vulnerable population subgroup, who may have higher exposure risks in terms of both non-carcinogenic and carcinogenic effect. Similarly, the highest HI of dieldrin for infants (2.26) in samples of processed cereal-based complementary food in Ghana was higher than the acceptable level [49]. The exposure of DDTs and HCHs in food in all age groups of females who are local residents from Nanjing, China coincided with the present study [50]. For OPPs residue, children are the most susceptible populations in rural residents exposed to chlorpyrifos in rice of China [51]. The adverse effects of malathion in grain from Kazakhstan adverse effects to women health was 3 times higher than men due to the alimentary, neurological, and respiratory system [52]. On the contrary, the study of the OPP pesticides poison risk in Thai men was greater than women [53]. Chronic exposure to chlorpyrifos and fenitrothion in South Australian children was commonly used in agriculture [54]. According to the European Union (EU) guidelines for OCP residues, the concentrations of DDTs (sum of *p,p’*-DDT, *o,p’*-DDT, *p-p’*-DDE, and *p,p’*- DDD), α-HCH, β-HCH, and γ-HCH in rice from Thailand exceeded the maximum residue limit (MRL), accounting for 7%, 26%, 26%, and 16% of total samples, respectively. Similarly, the residue levels of DDTs, α-HCH, and β-HCH in rice from China exceeded the MRL, accounting for 10%, 10%, and 30% of total samples, respectively (Table S5). According to the EU guidelines for OPP residues, the concentrations of methidathion in rice from Thailand and China exceeded the MRL, accounting for 21% and 40% of the total samples, respectively. The concentrations of phosalone in rice from Thailand and China exceeded the MRL, accounting for 21% and 40% of the total samples, respectively. However, the target pesticides of this study were below the MRL of China and Thailand standards [15,16,17,18] (Table S6). The concentrations of chlorpyrifos, diazinon, and pirimiphos-methyl were below the MRL, which coincided with rice from Iran [55]. 

## 5. Conclusions

The use of OCP pesticides has been banned several decades ago all over the world, despite this, OCPs residues are still found in rice and soils from Thailand and China. Farmers have mainly applied multiple types of pesticides to achieve greater efficiency in controlling pests and to increase productivity. The concentrations of ∑_22_OCPs were considerably higher than in the emerging substitutes, ∑_15_OPPs, in rice and soil samples from Thailand and China. The accumulation of OPPs in rice samples was susceptible compared to OCPs, which may be due to their lipophilicity, bioavailability, and mobility. Based on the bioconcentration factor, fatty content in rice grain could uptake pesticides from the contaminated soil by rhizosphere. Compared to literature data from other studies, the residue levels of OCPs from Thailand were in the level of moderate in rice and low in soil, whereas the residue level of OCPs in rice from China was low. The OPP levels in Thailand and China were low in rice and very low in soil.

According to the Chinese Soil Standard, DDT residues in soil from Thailand and China were low to moderately contaminated, whereas HCH residues in soil from Thailand and China were low. According to the EU guidelines for OCP residues in rice, the residue levels of OCPs from Thailand were moderately contaminated, whereas the residue levels of OCPs from China were low. The OPPs levels in Thailand and China were low in rice and very low in soil.

Based on the source evaluation of OCP residues, DDTs, endosulfans, and chlordane in soil from Thailand and China mainly originated from historical application. The relationship between TOC and all OCPs in soil had no significance, whereas a significant positive correlation between TOC and some OPPs in soil were exhibited (*p* < 0.05). The principal component analysis of OCPs showed no difference of OCPs residues in rice and soil between Thailand and China. In contrast, the PCA of OPPs of rice and soil from Thailand was significantly different from samples from China (*p* < 0.0083). 

The most sensitive population subgroups in term of OCP residues in rice are children in the vicinity of the paddy fields. The cooperation among farmers, individuals, non-profit organizations, local authorities, and government should be strengthened to eliminate pesticide residues, which may cause long-term health effects. Implementation of integrated pesticide management and good agricultural practices can contribute to the reduction of pesticide residues in paddy fields in the future. 

## Figures and Tables

**Table 1:** The sampling sites, number, and time of surface soil and rice from Thailand and China.

Location of Sampling Sites	Number of Paddy Soil	Number of Paddy Rice	Collection Time of Soil and Rice	Soil Texture
Khampaengphet, northern Thailand	10	10	April, 2015	silty clay loam
Nakorn Ratchasima, northeastern Thailand	5	5	September, 2015	sandy clay loam
Suratthani, southern Thailand	5	5	January, 2016	clay
Qingpu, Shanghai, China	3	3	February, 2016	clay
Sheyang, Jiansu, China	3	3	July, 2016	silty clay loam
Chongming Island, Shanghai, China	4	4	July, 2016	silty clay loam

**Table 2:** Hotelling’s T^2^-test comparing the relative concentration of OPPs in rice and soil from Thailand and China.

*p*-Value	RC	ST	SC	
Bonferroni (*p* < 0.0083) adjusted alfa (0.05)	0.0011 *	<0.0001 *	<0.0001 *	RT
		0.0094	<0.0001 *	RC
			0.0035 *	ST

RC (rice from China), RT (rice from Thailand), ST (soil from Thailand), SC (soil from China), * represented by *p* < 0.0083.

## Supplementary Materials

The following are available online at https://www.mdpi.com/1660-4601/17/11/3786/s1, Table S1. The OCP concentrations in rice from Thailand and China, Table S2. The OPP concentrations in rice from Thailand and China, Table S3. The OCP concentrations in soil from Thailand and China, Table S4. The OPP concentrations in soil from Thailand and China, Table S5. Maximum Residue Limit (MRL) for residues of organochlorine pesticides in rice under different guidelines, Table S6. Maximum Residue Limit (MRL) for residues of organophosphorus pesticides in rice under different guidelines.

## References

[1] Tilman D., Cassman K.G., Matson P.A., Naylor R., Polasky S. (2002). Agricultural sustainability and intensive production practices. Nature.

[2] 2018 Chinese Pesticide Exports Analysis-Southeast Asia. http://news.agropages.com/News/NewsDetail---29196.htm.

[3] Workman D. (2018). Top Pesticides Exporters. http://www.worldstopexports.com/top–pesticides–exporters/.

[4] Food and Agriculture Organization of the United Nations (2017). Pesticides: Pesticides-Average Use Per Area of Cropland. http://www.fao.org/faostat/en/?#data/.

[5] Ane N.U., Hussain M. (2015). Diversity of insect pests in major rice growing areas of the world. J. Entomol. Zool. Stud..

[6] Darko G., Acquaah S.O. (2008). Levels of organochlorine pesticides residues in dairy products in Kumasi, Ghana. Chemosphere.

[7] The Government of the Kingdom of Thailand (2007). Plan for the Implementation of Its Obligation under the Stockholm Convention on the Persistent Organic Pollutants (POPs) in Thailand.

[8] Nakata H., Hirakawa Y., Kawazoe M., Nakabo T., Arizono K., Abe S.K., Kitano T., Shimada H., Watanabe I., Li W. (2005). Concentrations and compositions of organochlorine contaminants in sediments, soils, crustaceans, fishes and birds collected from Lake Tai, Hangzhou Bay and Shanghai city region, China. Environ. Pollut..

[9] Hongsibsong S., Kerdnoi T., Polyiem W., Srinual N., Patarasiriwong V., Prapamontol T. (2018). Blood cholinesterase activity levels of farmers in winter and hot season of Mae Taeng District, Chiang Mai Province, Thailand. Environ. Sci. Pollut. Res..

[10] Gupta R.C., Mukherjee I.R.M., Doss R.B., Malik J.K., Milatovic D. (2017). Organophosphates and Carbamates. Reproductive and Developmental Toxicology.

[11] Wongta A., Sawarng N., Tongchai P., Sutan K., Kerdnoi T., Prapamontol T. (2018). The pesticide exposure of people living in agricultural community, Northern Thailand. J. Toxicol..

[12] Yimaer A., Chen G., Zhang M., Zhou L., Fang X., Jiang W. (2017). Childhood pesticide poisoning in Zhejiang, China: A retrospective analysis from 2006 to 2015. BMC Public Health.

[13] Michelangelo A., Steven J.L. (2003). Fast and Easy Multiresidue Method Employing Acetonitrile Extraction/Partitioning and “Dispersive Solid–Phase Extraction for the Determination of Pesticide Residue in Produce. J. AOAC Int..

[14] Khammanee N., He J., Qiu Y., Bignert A., Kungskulniti N., Niu D., Zhu Z. (2018). Occurrence of Organochlorine Pesticides and Polychlorinated Biphenyls in soil with Various Land Uses from Shanghai, China. Fresenius Environ. Bull..

[15] (2012). Food and Agriculture Organization of the United Nations - Joint FAO/WHO Meeting on Pesticide Residues. http://www.fao.org/fao–who–codexalimentarius/en/.

[16] Ministry of Agriculture and China Food and Drug Administration (2016). National Food Safety Standard (GB 2763–2016): Maximum Residue Limits for Pesticides in Food. https://www.chinesestandard.net/PDF/English.aspx/GB2763–2016.

[17] Ministry of Agriculture and Cooperatives Thailand and National Bureau of Agricultural Commodity and Food Standards (2016). Thai Agricultural Standard (TAS 9002–2016): Pesticide Residues: Maximum Residue Limits. https://www.acfs.go.th/#/fs.go.th/#/.

[18] European Commission (2016). Pesticide: Maximum Residue Limits. https://ec.europa.eu/food/plant/pesticides/max_residue_levels_en.

[19] Agency for Toxic Substances and Disease Registry (ATSDR) (1995). Public Health Assessment Guidance Manual.

[20] Lunney A., Zeeb B., Reimer K. (2004). Uptake of Weathered DDT in Vascular Plants: Potential for Phytoremediation. Environ. Sci. Technol..

[21] Chen S., Shi L., Shan Z., Hu Q. (2007). Determination of organochlorine pesticide residues in rice and human and fish fat by simplified two–dimensional gas chromatography. Food Chem..

[22] Nakata H., Kawazoe M., Arizono K., Abe S., Kitano T., Shimada H., Li W., Ding X. (2002). Organochlorine Pesticides and Polychlorinated Biphenyl Residues in Foodstuffs and Human Tissues from China: Status of Contamination, Historical Trend, and Human Dietary Exposure. Arch. Environ. Contam. Toxicol..

[23] Toteja G.S., Mukherjee A., Diwakar S., Singh P., Saxena B.N. (2003). Residues of DDT and HCH pesticides in rice samples from different geographical regions of India: A multicentre study. Food Addit. Contam..

[24] Chaiyarat R., Sookjam C., Eiam-Ampai K., Damrongphol P. (2015). Organochlorine pesticide levels in the food web in rice paddies of Bueng Boraphet wetland, Thailand. Environ. Monit. Assess..

[25] Suresh Babu G., Farooq M., Ray R.S., Joshi P.C., Viswanathan P.N., Hans R.K. (2003). DDT and HCH Residues in Basmati Rice (Oryza sativa) Cultivated in Dehradun (India). Water Air Soil Pollut..

[26] Mumtaz M., Qadir A., Mahmood A., Mehmood A., Malik R.N., Li J., Yousef Z., Jamil N., Shaikh L.A., Ali H. (2015). Human health risk assessment, congener specific analysis and spatial distribution pattern of organochlorine pesticides (OCPs) through rice crop from selected districts of Punjab Province, Pakistan. Sci. Total Environ..

[27] Mahmood A., Malik R.N., Li J., Zhang G. (2014). Human health risk assessment and dietary intake of organochlorine pesticides through air, soil and food crops (wheat and rice) along two tributaries of river Chenab, Pakistan. Food Chem. Toxicol.

[28] Hoai P.M., Sebesvari Z., Minh T.B., Viet P.H., Renaud F.G. (2011). Pesticide pollution in agricultural areas of Northern Vietnam: Case study in Hoang Liet and Minh Dai communes. Environ. Pollut..

[29] Yadav I.C., Devi N.L., Li J., Zhang G., Shakya P.R. (2016). Occurrence, profile and spatial distribution of organochlorines pesticides in soil of Nepal: Implication for source apportionment and health risk assessment. Sci. Total Environ..

[30] Zhou Q., Wang J., Meng B., Cheng J., Lin G., Chen J., Zheng D., Yu Y. (2013). Distribution and sources of organochlorine pesticides in agricultural soils from central China. Ecotoxicol. Environ. Saf..

[31] Mishra K., Sharma R.C., Kumar S. (2012). Contamination levels and spatial distribution of organochlorine pesticides in soils from India. Ecotoxicol. Environ. Saf..

[32] Guo W., Zhang H., Huo S. (2014). Organochlorine pesticides in aquatic hydrophyte tissues and surrounding sediments in Baiyangdian wetland, China. Ecol. Eng..

[33] Liu W.-X., He W., Qin N., Kong X.-Z., He Q.-S., Ouyang H.-L., Yang B., Wang Q.-M., Yang C., Jiang Y.-J. (2012). Residues, Distributions, Sources, and Ecological Risks of OCPs in the Water from Lake Chaohu, China. Sci. World J..

[34] Science and Technology Department of State Environmental Protection (1995). Administration Environmental Quality Standard for Soils (GB15618–1995).

[35] Snyder F., Ni L. (2017). A Tale of Eight Pesticides: Risk Regulation and Public Health in China. Eur. J. Risk Regul..

[36] Washburn A.D. (2003). The Environmental Fate of Methidathion. https://www.cdpr.ca.gov/docs/emon/pubs/fatememo/methidathion.

[37] Manirakiza P., Akinbamijo O., Covaci A., Pitonzo R., Schepens P. (2003). Assessment of Organochlorine Pesticide Residues in West African City Farms: Banjul and Dakar Case Study. Arch. Environ. Contam. Toxicol..

[38] Miglioranza K.S.B., Aizpún de Moreno J.E., Moreno V.J., Osterrieth M.L., Escalante A.H. (1999). Fate of organochlorine pesticides in soils and terrestrial biota of “Los Padres” pond watershed, Argentina. Environ. Pollut..

[39] Stadlinger N., Berg H., Van den Brink P.J., Tam N.T., Gunnarsson J.S. (2016). Comparison of predicted aquatic risks of pesticides used under different rice–farming strategies in the Mekong Delta, Vietnam. Environ. Sci. Pollut. Res..

[40] Ghanbari F., Monavari F., Monavari S.M., Arjmandi R. (2017). Human health risk assessment of organophosphorus pesticide in rice crop from selected districts of Anzali International Wetland basin, Iran. Hum. Exp. Toxicol..

[41] Bajet C.M., Tejada A.W. (1995). Pesticide residues in the Philippines: An analytical perspective. Trends. Analyt. Chem..

[42] Pan H.W., Lei H.J., He X.S., Xi B.D., Xu Q.G. (2019). Spatial distribution of organochlorine and organophosphorus pesticides in soil–groundwater systems and their associated risks in the middle reaches of the Yangtze River Basin. Environ. Geochem. Health.

[43] Zhang X., Shen Y., Yu X.-Y., Liu X.-J. (2012). Dissipation of chlorpyrifos and residue analysis in rice, soil and water under paddy field conditions. Ecotoxicol. Environ. Saf..

[44] Tri J., Sutrisno A., Henna R.S., Savitri R. (2017). Pesticides Usage in the Soil Quality Degradation Potential in Wanasari Subdistrict, Brebes, Indonesia. Appl. Environ. Soil Sci..

[45] Feng K., Yu B.Y., Wang X.L., Ge D.M., Wang X.Z., Wong M.H., Cao Z.H. (2004). Distribution of Organo–Chlorine Pesticides (DDT and HCH) Between Plant and Soil System. Environ. Geochem. Health.

[46] Han Y., Mo R., Yuan X., Zhong D., Tang F., Ye C., Liu Y. (2017). Pesticide residues in nut–planted soils of China and their relationship between nut/soil. Chemosphere.

[47] Fang Y., Nie Z., Yang J., Die Q., Tian Y., Liu F., He J., Wang J., Huang Q. (2018). Spatial distribution of and seasonal variations in endosulfan concentrations in soil, air, and biota around a contaminated site. Ecotoxicol. Environ. Saf..

[48] Jiang Y.-F., Wang X.-T., Jia Y., Wang F., Wu M.-H., Sheng G.-Y., Fu J.-M. (2009). Occurrence, distribution and possible sources of organochlorine pesticides in agricultural soil of Shanghai, China. J. Hazard. Mater..

[49] Akoto O., Oppong-Otoo J., Osei-Fosu P. (2015). Carcinogenic and non–carcinogenic risk of organochlorine pesticide residues in processed cereal–based complementary foods for infants and young children in Ghana. Chemosphere.

[50] Zhang Q., Xia Z., Wu M., Wang L., Yang H. (2017). Human health risk assessment of DDTs and HCHs through dietary exposure in Nanjing, China. Chemosphere.

[51] Zhang C.Z., Zhang X.M., Tian Z.H., He D.J., Liu X.J. (2010). Degradation of Chlorpyrifos and Fipronil in rice from farm to fork and risk assessment. Agr. Sci. China.

[52] Lozowicka B., Kaczynski P., Paritova A.E., Kuzembekova G.B., Abzhalieva A.B. (2014). Pesticide residues in grain from Kazakhstan and potential health risks associated with exposure to detected pesticides. Food Chem. Toxicol..

[53] Tawatsin A., Thavara U., Siriyasatien P. (2015). Pesticides Used in Thailand and Toxic Effects to Human Health. Med. Res. Arch..

[54] Babina K., Dollard M., Pilotto L., Edwards J.W. (2012). Environmental exposure to organophosphorus and pyrethroid pesticides in South Australian preschool children: A cross sectional study. Environ. Int..

[55] Maryam A., Hassan Y., Fazad K., Shahram S., Morteza P.-H., Hossein R. (2018). Exposure Assessment for Some Pesticides through Rice Consumption in Iran Using a Multiresidue Analysis by GC–MS. Iran. J. Pharm. Res..

