# Supplementary material for: Presence and Health Risks of Obsolete and Emerging Pesticides in Paddy Rice and Soil from Thailand and China
Source: Int J Environ Res Public Health. 2020 May 27;17(11):3786. doi: 10.3390/ijerph17113786 (PMC7312988; doi:10.3390/ijerph17113786)
Supplement: Supplementary file 1 [file ijerph-17-03786-s001.pdf]

Supplementary Materials

Table S1. The OCP concentrations in rice from Thailand and China.

OCPs	Thailand (n=20)(ngg^{-1})			China (n=10) (ngg^{-1})		
	Range	Mean	Median	Range	Mean	Median
∑HCHs	n.d. - 1440	377	30.2	0.76 - 250	40.8	8.06
∑DDTs	3.92 - 257.	59.6	32.3	4.82 - 177	37.0	21.5
∑CHLs	2.95 - 163	25.7	11.2	0.22 - 21.6	7.47	4.55
∑Drins	4.22 - 151	41.6	21.4	n.d. - 7.73	2.50	1.86
∑Endosulfans	n.d. - 7.73	2.50	1.86	n.d. - 4.50	1.34	1.19
HCB	n.d. - 25.0	4.44	0.86	n.d. - 3.64	0.79	0.12
methoxychlor	n.d. – 40.3	10.6	5.54	n.d. - 10.6	3.53	3.41
mirex	n.d. - 29.5	6.89	4.61	0.79 - 50.9	8.11	3.39
$\sum_{22}$OCPs	34.0 - 2061	528	107	37.4 – 393	102	44.0

Note: n.d. (non-detectable)

Table S2. The OPP concentrations in rice from Thailand and China.

OPPs	Thailand (n=20)(ngg^{-1})			China (n=10) (ngg^{-1})		
	Range	Mean	Median	Range	Mean	Median
Dichlorvos	n.d.	n.d.	n.d.	n.d.	n.d.	n.d.
Fonofos	n.d. - 0.38	0.12	0.11	n.d. - 0.69	0.28	n.d.
Diazinon	n.d. - 5.74	1.51	1.22	n.d. - 4.03	1.35	0.97
Chlorpyrifos -methyl	n.d. - 0.75	0.05	n.d.	n.d.	n.d.	n.d.
Methyl parathion	n.d. - 3.63	0.19	n.d.	n.d.	n.d.	n.d.
Pirimiphos-methyl	n.d. - 0.91	0.51	0.91	n.d. - 0.91	0.82	0.91
Fenitrothion	0 - 0.21	0.07	0.09	n.d.	n.d.	n.d.
Azinphos methyl	n.d. - 0.70	0.39	0.7	n.d. - 4.47	1.48	1.45
Malathion	n.d. - 0.78	0.15	n.d.	n.d. - 0.08	0.02	n.d.
Chlorpyrifos	n.d. - 75.37	9.38	n.d.	n.d. - 68.9	9.77	1.79
Parathion	n.d. - 1.67	0.10	n.d.	n.d.	n.d.	n.d.
Methidathion	n.d. - 138	20.4	7.26	1.25 - 78.8	32.7	19.8
Ethion	n.d. - 10.7	2.30	0.18	n.d. - 1.51	0.32	0.05
Carbophenothion	n.d. - 38.3	6.43	5.43	n.d. - 6.27	2.3	2.48
Phosalone	n.d. - 25.2	5.64	1.10	n.d. - 53.1	12.0	0.10
∑15OPPs	5.14 - 171	47.2	17.0	19.96 - 118	61.0	27.6

Note: n.d. (non-detectable)

Table S3. The OCP concentrations in soil from Thailand and China.

OCPs	Thailand (n=20)(ngg^{-1})			China (n=10) (ngg^{-1})		
	Range	Mean	Median	Range	Mean	Median
∑HCHs	0.06 - 536	100	8.10	2.86 - 190.	53.4	37.2
∑DDTs	3.64 - 120	29.0	16.8	18.9 - 88.4	44	40.6
∑CHLs	2.34 - 23.3	9.27	7.00	3.11 - 19.3	10.3	7.54
∑Drins	4.37 - 145.	28.6	11.5	7.19 - 97.1	32.6	21.2
∑Endosulfans	n.d. - 9.52	1.65	0.64	n.d. - 9.47	5.49	4.69
HCB	n.d. - 22.6	3.15	1.13	n.d. - 21.0	4.00	4.69
methoxychlor	n.d. - 74.5	9.11	3.51	n.d. - 12	3.19	1.84
mirex	0.80 - 49.1	7.80	3.38	n.d. - 25.5	7.39	3.85
$\sum_{22}$OCPs	3.37 – 135	189	52.1	55.2 – 407	160	121

Note: n.d. (non-detectable)

Table S4. The OPP concentrations in soil from Thailand and China.

OPPs	Thailand (n=20)(ngg^{-1})			China (n=10) (ngg^{-1})		
	**Range**	**Mean**	**Median**	**Range**	**Mean**	**Median**
Dichlorvos	n.d.	n.d.	n.d.	n.d.	n.d.	n.d.
Fonofos	n.d. - 0.11	0.04	0.01	n.d. - 0.19	0.07	0.05
Diazinon	n.d. - 11.0	1.01	0.22	0.13 - 20.9	2.92	1.09
Chlorpyrifos -methyl	n.d. - 0.09	0.05	0.09	n.d. - 0.09	0.05	0.09
Methyl parathion	n.d.	n.d.	n.d.	n.d. - 0.93	0.09	n.d.
Pirimiphos-methyl	n.d. - 0.91	0.55	0.91	n.d. - 0.15	0.07	0.08
Fenitrothion	n.d. - 0.13	0.03	n.d.	n.d.	n.d.	n.d.
Azinphos methyl	n.d. - 2.90	1.14	0.70	n.d.	n.d.	n.d.
Malathion	n.d. - 0.08	0.05	0.07	n.d. - 0.26	0.12	0.17
Chlorpyrifos	n.d. - 44.5	7.09	0.41	n.d. - 0.91	0.24	0.24
Parathion	n.d. - 0.13	0.04	n.d.	n.d. - 0.39	0.14	0.07
Methidathion	0.23 - 16.5	3.52	1.30	n.d. - 1.30	1.04	1.30
Ethion	n.d. - 0.55	0.05	n.d.	n.d. - 0.97	0.11	n.d.
Carbophenothion	n.d. - 8.39	1.14	n.d.	n.d. - 4.54	0.68	n.d.
Phosalone	n.d. - 0.22	0.11	0.16	n.d. - 0.16	0.04	n.d.
∑15OPPs	2.05 - 58.7	14.8	3.87	2.91 - 22.2	5.57	3.09

Note: n.d. (non-detectable)

Table S5. Maximum Residue Limit (MRL) for residues of organochlorine pesticides in rice under different guidelines.

Pesticides	MRL of EU[18] (mg.kg^{-1})	MRL of China[16] (mg.kg^{-1})	MRL of Thailand[17] (mg.kg^{-1})
Aldrin	0.01 (Aldrin + dieldrin)	0.02	0.02 (Aldrin + dieldrin)
p,p′-DDD<br>*p,p′*-DDE<br>*p,p′*-DDT<br>o,p′-DDT	0.05	0.1	0.1
Dieldrin	-	0.02	-
Endrin	0.01	0.01	0.01
Endosulfans	0.05	-	-
Hexachloro benzene (HCB)	0.01	-	-
α–HCH	0.01		
β–HCH	0.01		
γ–HCH (lindane)	0.01	0.05	-
δ-HCH	-		
Methoxychlor	0.01	-	-
Mirex	-	-	-
Heptachlor	-	-	0.02

Note: No available data for rice is depicted by "-" in the table.

Table S6. Maximum Residue Limit (MRL) for residues of organophosphorus pesticides in rice under different guidelines.

Compounds	MRL of EU[18] (mg.kg-1)	MRL of China[16] (mg.kg-1)	MRL of Thailand[17] (mg.kg-1)
Azinphos methyl	0.05	-	-
Carbophenothion	-		-
Chlorpyrifos	0.5	0.5	0.1
Chlorpyrifos methyl	3	5	-
Diazinon	0.01	0.1	-
Dichlorvos	0.01	0.1	-
Ethion	0.01	0.2	-
Fenitrothion	0.05	5	1
Fonofos	-	0.05	-
Malathion	8	8	0.05
Methidathion	0.02	0.05	-
Parathion-methyl	0.02	0.2	-
Parathion	0.05	0.1	-
Phosalone	0.01	-	-
Pirimiphos-methyl	0.5	5	5

Note: No available data for rice is depicted by "-" in the table.
